# Ready Estimation of Fat in Milk

**Published:** 1875-03

**Authors:** 


Ready Method of Estimating the Fat (Cream) Present in Milk.—John Horsley, F.C.S., (*Chemical News*) describes a simple method of determining the amount of cream in any sample of milk, as follows: The milk is agitated in a graduated glass tube, with its bulk of ether, for four or five minutes. Alcohol is then added, equal in volume to the milk employed, and the whole well shaken together for five minutes. The tube is then placed in a a vertical position, and allowed to remain at rest, when the oily matter will quickly rise to the surface, and its amount can be read off at once on the scale, and the percentage thence readily computed.—*Detroit Review of Medicine and Pharmacy.*

